# Coordination of Cellular Dynamics Contributes to Tooth Epithelium Deformations

**DOI:** 10.1371/journal.pone.0161336

**Published:** 2016-09-02

**Authors:** Ritsuko Morita, Miho Kihira, Yousuke Nakatsu, Yohei Nomoto, Miho Ogawa, Kazumasa Ohashi, Kensaku Mizuno, Tetsuhiko Tachikawa, Yukitaka Ishimoto, Yoshihiro Morishita, Takashi Tsuji

**Affiliations:** 1 Research Institute for Science and Technology, Tokyo University of Science, Noda, Chiba, 278-8510, Japan; 2 Department of Biological Science and Technology, Graduate School of Industrial Science and Technology, Tokyo University of Science, Katsushika-ku, Tokyo, 125-8585, Japan; 3 Department of Biomolecular Sciences, Graduate School of Life Sciences, Tohoku University, Sendai, Miyagi, 980-8578, Japan; 4 Department of Oral Pathology, Showa University School of Dentistry, Shinagawa-ku, Tokyo, 142-8555, Japan; 5 Department of Machine Intelligence and Systems Engineering, Akita Prefectural University, Yurihonjo, Akita 015-0055, Japan; 6 Laboratory for Developmental Morphogeometry, RIKEN Quantitative Biology Center, Kobe 650-0047, Japan; Laboratoire de Biologie du Développement de Villefranche-sur-Mer, FRANCE

## Abstract

The morphologies of ectodermal organs are shaped by appropriate combinations of several deformation modes, such as invagination and anisotropic tissue elongation. However, how multicellular dynamics are coordinated during deformation processes remains to be elucidated. Here, we developed a four-dimensional (4D) analysis system for tracking cell movement and division at a single-cell resolution in developing tooth epithelium. The expression patterns of a Fucci probe clarified the region- and stage-specific cell cycle patterns within the tooth germ, which were in good agreement with the pattern of the volume growth rate estimated from tissue-level deformation analysis. Cellular motility was higher in the regions with higher growth rates, while the mitotic orientation was significantly biased along the direction of tissue elongation in the epithelium. Further, these spatio-temporal patterns of cellular dynamics and tissue-level deformation were highly correlated with that of the activity of cofilin, which is an actin depolymerization factor, suggesting that the coordination of cellular dynamics via actin remodeling plays an important role in tooth epithelial morphogenesis. Our system enhances the understanding of how cellular behaviors are coordinated during ectodermal organogenesis, which cannot be observed from histological analyses.

## Introduction

The three-dimensional (3D) morphologies of ectodermal organs are required for their inherent physiological and physical functions and are formed by an accumulation of highly dynamic cell behaviors during embryonic development [1–3]. Spatiotemporal regulation and the combination of epithelial deformation modes, including invagination, lumen formation, branching, and anisotropic tissue expansion/elongation, determine the final organ shape [1, 2, 4, 5]. The molecular genetic approach has revealed dozens of genes that are involved in the regulation of epithelial tissue formation. The roles of morphogens and those of the cytoskeleton and cell adhesion molecules have been elucidated primarily at the cellular level [6]. The actin cytoskeleton that is coordinated by actin-depolymerizing factor (ADF)/cofilin has been implicated in cell shape, motility and proliferation in response to external stimuli and intracellular signals [6–8]. Previous studies have indicated the importance of actin reorganization for gastrulation and eye cup formation via the actin-myosin network and signal transduction through small G proteins [6, 7, 9, 10]. However, how the spatiotemporal changes in cellular behavior and signaling events cooperate to regulate local deformations and contribute to the overall 3D organ shape during dynamic morphogenetic processes remains unknown. To understand the regulatory mechanisms underlying organ morphogenesis, it is essential to visualize and track the cellular dynamics as well as the signaling events at a single-cell resolution [11].

Ectodermal organs, such as the teeth and hair, undergo several morphological changes during early development [2, 12]. After the formation of primordial germ cells and associated epithelium thickening via reciprocal interactions between the epithelium and the underlying mesenchyme, the epithelium invaginates into the underlying mesenchyme to form a bud shape. Then, the bud folds at its tip and forms a cap-like structure covering the underlying mesenchyme. This bud-to-cap transition of the epithelium is not only a common folding deformation phenomenon for both the teeth and hair but is also a crucial checkpoint for subsequent developmental processes, such as the differentiation of specialized cells and the determination of the final shape and size of the organ [13]. In tooth development, the continuous elongation of the folding epithelium and the growth of the underlying mesenchyme result in a bell-shaped epithelium with a tooth crown that evoking the final shape of the tooth [1, 13, 14]. Recent studies have demonstrated that in the late bud stage, the enamel knot (EK), which consists of non-dividing cells, emerges at the tip of the bud where the folding of the epithelium starts, and it functions as a signaling center through the expressions of many signaling molecules that induce tooth morphogenesis and differentiation [1, 13, 14]. Although the genetic regulation involved in morphogenesis is being uncovered, the cellular mechanisms that drive the cap and bell shape formation have remained largely unanalyzed.

To elucidate the inter-hierarchical relationships between tissue deformation, cellular behaviors and regulatory molecules in tooth morphogenesis, it is important to develop a long-term cell tracking system that allows multiple cells to be visualized in growing tissues at the single-cell resolution [11]. Recent innovations in microscopy and the development of attractive fluorescein probes have enabled us to visualize and record a series of complex morphogenetic processes at multiple scales ranging from macro-imaging of tissue shape changes to micro-imaging of individual cell behaviors, protein dynamics and changes in gene expression [11, 15, 16]. Previous studies have primarily focused on cell behaviors in two-dimensional (2D) epithelial sheets that transform into three-dimensional (3D) structures [17–22]. However, the single-cell resolution analysis of 3D organs, such as mouse teeth, remains challenging despite the ability to culture ectodermal organs *ex vivo* because the interior of the embryonic tooth germ is difficult to access.

In our current study, we established a four-dimensional (4D) analysis system for the long-term visualization of the developing tooth germ at a single-cell resolution. We demonstrated that at the tissue level, the spatiotemporal pattern of the epithelium deformation in the tooth germ was determined by a combination of several basic tissue deformation modes, including invagination, anisotropic flattening and directional elongation. At the cellular level, this process was achieved by the accurate regulation of the spatiotemporal patterns of cell proliferation, division orientation, and motility. At the molecular level, we found that the pattern of cofilin activity exhibited a strong correlation with that of the cellular dynamics, which implies that actin remodeling is a key regulator of cell dynamics at the higher cell/tissue scales. Our multi-scale analyses enabled us to bridge the gap between cell behaviors and tissue shape changes during the tooth development process and enhance our knowledge of the inter-hierarchical relationships between cellular behaviors and tissue/organ morphologies.

## Materials and Methods

### Animals

C57BL/6 mice were purchased from SLC, Inc. (Shizuoka, Japan). B6.B6D2-Tg(Fucci)504Bsi mice and B6.B6D2-Tg(Fucci)596Bsi mice were obtained from the RIKEN Bioresource Center (Chiba, Japan) [16]. R26-H2B-EGFP mice were kindly provided by Dr. Toshihiko Fujimori (National Institute for Basic Biology, Aichi, Japan) [23]. All mouse care and handling complied with the NIH guidelines for animal research. All experimental protocols were approved by the Tokyo University of Science Animal Care and Use Committee.

### 4D live imaging of the organ germ

Live imaging was performed using a confocal microscope (LSM780; Carl Zeiss, Oberkochen, Germany) combined with a CO2 incubator. The pregnant mice were euthanized by cervical dislocation, and the molar tooth germs and whisker follicles were then dissected from the mandibles and maxilla of embryonic days (E) 12.5–13.5 mice. The anterior and posterior parts of the molar tooth germs were cut off with needles, and the remaining intermediate region (250–300-μm-thick frontal slices) were used for culture (S1 Video). The germs were fixed in a drop of collagen type I-A (Nitta gelatin, Osaka, Japan) on a 35-mm glass-bottomed plastic dish (Iwaki, Tokyo, Japan) coated with collagen type I-P (Nitta gelatin) and were then immersed in Dulbecco’s modified Eagle’s medium (DMEM) supplemented with 10% fetal bovine serum (GIBCO, Grand Island, NY, USA), 100 μg/ml ascorbic acid (Sigma, St. Louis, MO, USA), and 2 mM L-glutamine (Sigma). For high-resolution live imaging, stacks of optical section images (1024×1024 pixels for the x-y plane and 2 μm for the z-axis step, 90–100 slices) were acquired at 30-min intervals over 5 days using a 25× oil-immersion lens (N.A. 0.8, Zeiss).

### 4D cell tracking analysis

For the analysis of cell behavior, the confocal images were processed with Imaris 7.4 (Bitplane, Inc., Saint Paul, MN, USA). The cells were tracked manually with GFP-labeled histone 2B (H2B), which is uniformly present in the nuclei of all cells, enabling observation of cell division throughout the development of the germs. To avoid the influence of the tissue repair response on this analysis, we excluded the tracking data from a 30-μm-deep layer from the cut surface as analysis objects because excess cell proliferation was often observed on the cut surface (S2A and S2B Fig). The 3D epithelial representations were generated by manually drawing a boundary line between the epithelium and the mesenchyme using Imaris. The epithelium and mesenchyme in the tooth germ are spatially separated by a basement membrane consisting of extracellular matrix. The peripheral cells of the epithelium align on the boundary between the epithelium and mesenchyme by making a contact with the basement membrane. The border between the epithelium and mesenchyme was distinguished based on the black-edged aligned epithelial layer (the white arrowhead in S2C Fig, left-lower panel).

### Analysis of cell division orientation

The three-dimensional x-y-z axis coordinates of the two daughter cell nuclei were obtained in the series of z-stack images with Imaris, and the division angles were calculated using inverse trigonometric functions.

### Deformation analysis

Tissue deformation maps over 5-hour periods during different stages corresponding to E13.5, E14.5, E15.5, and E16.5 were constructed based on the 3D cell trajectory data by applying the method proposed by Morishita and Suzuki [24]. In this method, a regular lattice enveloping the target organ before deformation is considered, and the lattice deformation over a given time interval is estimated (S3B Fig). Because the cultured tooth germ is a three-dimensional structure, we first checked how the two-dimensional deformation patterns on the x-y plane spanned the buccal-lingual axis and the oral-aboral axis depending on the position along the “z-direction” (i.e., the anterior-posterior axis). To achieve this, we estimated 2D maps for each of the 40-μm-thick z-slices. Consequently, the spatiotemporal patterns of the typical deformation characteristics, volume growth rate and deformation anisotropy were very similar among the z-slices (S4 Fig). We concluded that the tissue deformation dynamics could be approximately regarded as 2D-like during the period assessed. Finally, we constructed 2D maps using all of the cell position data after projecting those data into an x-y plane. The positional data of approximately 400 cells were used to estimate each map. The spatiotemporal patterns of the volume growth rate and deformation anisotropy were calculated from the constructed 2D maps.

### Adenovirus

Plasmids encoding YFP-human wild-type LIM-Kinase (YFP-hLIMK WT), YFP-hLIMK D460A, YFP-hRac, and YFP-Actin were provided by K. Mizuno (Tohoku University, Japan). Adenoviral vectors carrying the above-mentioned genes were constructed with an Adenovirus Cre/loxP Kit (TaKaRa Bio Inc., Shiga, Japan), and the recombinant adenoviruses were generated according to the manufacturer's protocol.

### Photobleaching assay

Photobleaching of the YFP-Actin was performed with a 488-nm laser at 100% transmission for 100 iterations using a laser scanning confocal microscope (LSM780; Carl Zeiss) equipped with a 40× Plan-Apochromat objective lens (NA 1.4). Fluorescent recovery after photobleaching was observed 200 times every 500 ms. The signal at the first post-bleach time was subtracted from all of the post-bleach measurements, the percentage of fluorescence recovered at each time point was calculated as the difference between the pre-bleach and first post-bleach measurements, and the half-time for recovery was estimated.

### Western blotting

Non-fixed cryosections (10 μm) were prepared and dried before microdissection. The particular areas in the tissues were obtained by laser microdissection (PALM microlaser system, Carl Zeiss) and collected separately onto 500-μl opaque adhesive caps (P.A.L.M. Microlaser Technologies). The microdissected tissues were lysed with 10 μl sample buffer. The lysates were boiled at 99°C, and the samples were subjected to SDS–PAGE (precast IEF pH 3–10 gels were from Bio-Rad, Hercules, CA, USA). After PAGE, the proteins were transferred to polyvinylidene difluoride membranes and revealed by immunostaining with the following primary antibodies: Cofilin (rabbit anti-cofilin Ab; 1:1000, gift from Dr. Mizuno); p-Cofilin (rabbit anti-phosphorylated cofilin Ab; 1:1000, gift from Dr. Mizuno); and GAPDH (rabbit anti-GAPDH Ab; 1:1000, Cell Signaling Technology, Beverly, MA). The primary antibodies were detected using HRP-conjugated goat anti-rabbit IgG (1:5000, Cappel Laboratories, Cochranville, PA).

### Histochemical analysis and immunohistochemistry

Histochemical tissue analyses were performed as previously described [25, 26]. Tissue sections (5–10 μm) were stained with hematoxylin-eosin and observed using Axioimager A1 (Carl Zeiss) with AxioCAM MRc5 (Carl Zeiss) microscopes.

For tissue preparation for immunohistochemistry, the mice were transcardially perfused with 4% paraformaldehyde in PBS under deep anesthesia with sodium pentobarbital (50 mg/kg, i.p.). The tissues were then removed and further post-fixed for 2–16 hours at 4°C. After fixation, the tissues were prepared as previously described [25, 26]. For fluorescent immunohistochemistry, the sections (35 μm) were incubated with the following primary antibodies: Cofilin (rabbit anti-cofilin Ab; 1:100, a gift from Dr. Mizuno); p-Cofilin (rabbit anti-phosphorylated cofilin Ab; 1:100, a gift from Dr. Mizuno); Ki67 (rabbit anti-Ki67 Ab; 1:100, Abcam, Cambridge, MA); Nidogen (rat anti-Nidogen Ab; 1:200, Millipore, Billerica, MA); Alexa Fluor 633 Phalloidin (1:50, Molecular Probes, Eugene, OR); and Hoechst 33342 (1:500, Molecular Probes). The primary antibodies were detected using the following secondary antibodies: Alexa Fluor 488-conjugated goat anti-rabbit IgG (1:200, Molecular Probes); Alexa Fluor 594-conjugated goat anti-rabbit IgG (1:200, Molecular Probes) and Alexa Fluor 555-conjugated goat anti-rat IgG (1:200, Molecular Probes). All fluorescence microscopy images were captured with a confocal microscope (LSM780; Carl Zeiss).

### *In situ* hybridization

The *in situ* hybridizations were performed using 10-μm frozen sections as previously described [25, 26]. Digoxigenin-labeled probes for specific transcripts were prepared by PCR with primers designed using published sequences (GenBank Accession Numbers; *LIM-Kinase 1* (*Limk1*): NM_010717, *LIM-Kinase 2* (*Limk2*): NM_001034030, *Testis-Specific Kinase 1* (*Tesk1*): NM_011571, *Slingshot 1*: NM_198109, *Slingshot 2*: NM_177710, and *Slingshot 3*: NM_198113).

### Cell proliferation and wound healing assay

The molar epithelial tissues were dissected from the mandibles of E14.5 mice and diced into fine pieces using a fine needle. The diced tissues were seeded in collagen-coated dishes and incubated for 24 hours. The cells were transfected with adenovirus containing wild-type or mutant LIMK. The cell proliferation ratio was determined by measuring the numbers of living cells after 24 hours and 60 hours of transfection using the Cell Counting Kit-8 (Dojindo Labs, Tokyo, Japan) according to the manufacturer's protocol. To measure cell migration, a wound was introduced in the outgrowth area of the epithelial tissues using a pipette tip after 24 hours of transfection. The cell migration over 12 hours was measured.

### Statistics

P values were calculated using Student’s *t*-tests. *P < 0.01 and * * 0.001 < *P* < 0.005.

## Results

### Spatial-temporal cell growth during tooth morphogenesis

**Fig 1:**
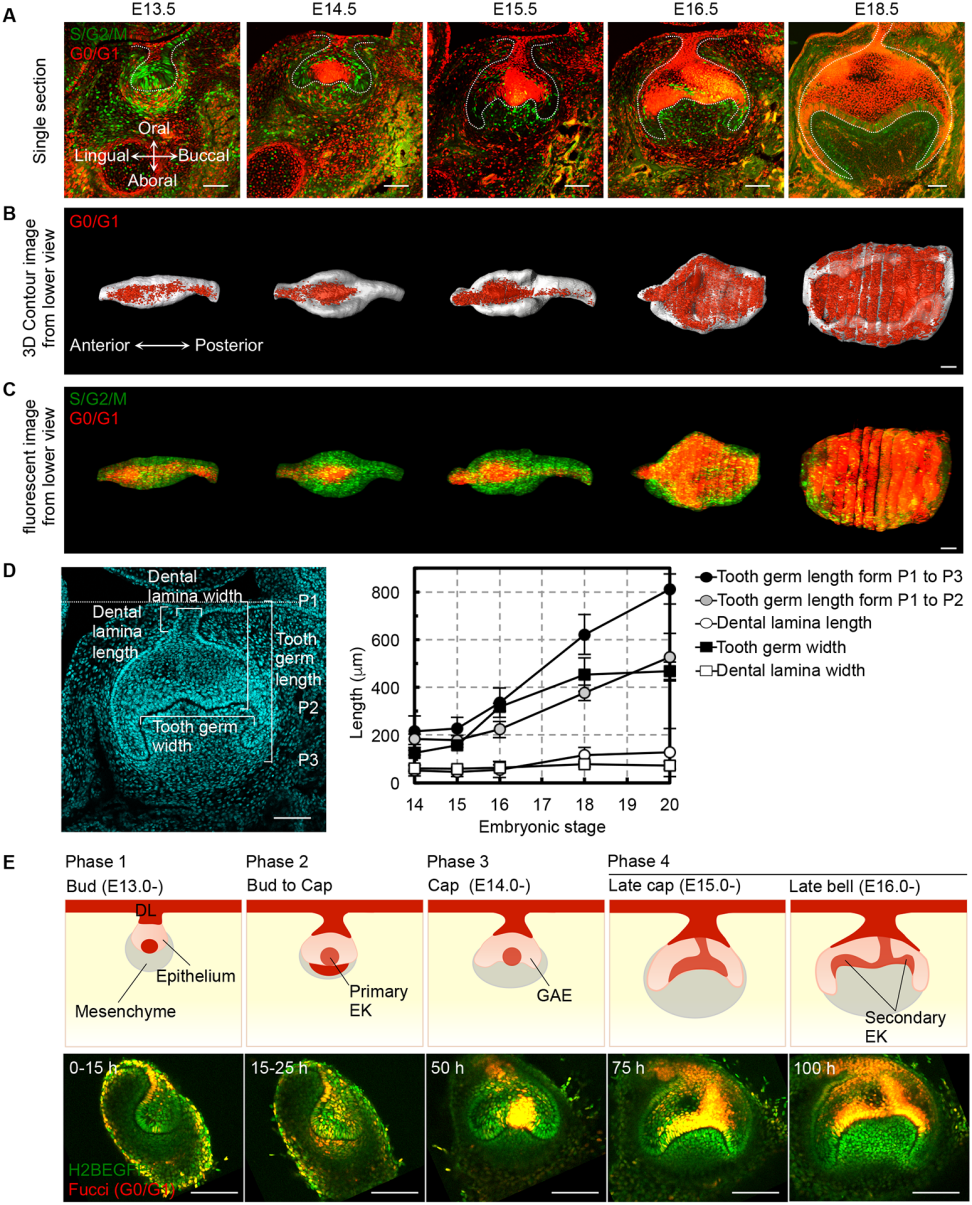
Tooth morphogenesis and spatial-temporal cell proliferation. **(A)** Frontal sections of mandibular molar tooth germ derived from Fucci mice at embryonic day (E) 13.5–18.5. The lingual side is on the left in all panels. The scale bars represent 100 μm. **(B)** Three-dimensional volume rendering images of the molar epithelium shown from the jaw side. The scale bars represent 100 μm. **(C)** Three-dimensional reconstructions of frontal sections of Fucci tooth germ epithelia showing the cell proliferation pattern. The scale bars represent 100 μm. **(D)** Length and width measurements of each part of the molar tooth germ. The results are provided as the mean ± s.d. of 6 samples (E14), 6 samples (E15), 8 samples (E16), 7 samples (E18) and 4 samples (E20). The measurement parts are indicated in the left figure. The scale bars represent 100 μm. **(E)** *Ex vivo* imaging of four phases during tooth germ epithelium morphogenesis. Schematic (upper panels) and captured live images (lower panels) are provided. The lingual side is on the left in all panels. Red indicates the growth-arrested regions. DL, dental lamina; EK, enamel knot; GAE, growing apex of epithelium. The scale bars represent 100 μm.

Organ morphology is shaped by diverse collective cell behaviors [21]. In tooth morphogenesis, it is known that cell proliferation is strictly controlled in each morphological process, such as epithelial invagination, elongation and bell-shaped formation [14]. Thus, we first validated the expression pattern of the Fucci probe, which allows for the clear visualization of the cell cycle phases of S/G_2_/M and G_0_/G_1_ with mAG-hGeminin and mKO2-hCdt1 fluorescence, respectively, in the developing tooth germ from embryonic days (E) 13.5 to 18.5 (Fig 1A–1C and S1 Fig). As previously reported, growth-arrested epithelial cell populations were first observed in the dental lamina and then in the tip of the invaginated epithelium at the bud stage at E13.5. Growth arrest was also observed in the primary EK, which is an important signaling center in tooth development, and in a secondary EK that is involved in cusp formation (Fig 1A–1C and S1 Fig). The growth-arrested region extended over the entire dental epithelium with the exception of the inner enamel epithelium and the cervical loop tips between E15.5 and 18.5 after bell-shape formation (Fig 1A–1C and S1 Fig).

To further analyze the relationship between tooth size and spatial-temporal cell proliferation during tooth morphogenesis, we also measured the lengths of various tooth germ regions from E14 to E20. In the dental lamina, which is a growth-arrested region at these stages, the length and width did not change (Fig 1D). The tooth germ width was also unchanged after the bell stage at E18. In contrast, the tooth germ length continuously elongated according to the embryonic stage between E14 to E20. These profiles of tooth germ size changes during tooth crown and root development exhibited a strong high correlation with the spatiotemporal pattern of epithelial cell proliferation illustrated by the Fucci probe.

To visualize these tooth morphogenetic dynamics sequentially, we established an *ex vivo* time-lapse imaging system using transgenic mouse embryos co-expressing the Fucci-G0/G1 probe (Cdt1-mKO) and enhanced green fluorescent protein-labeled human histone 2B (H2B-EGFP; S1 and S2 Videos). The transgenic mouse-derived germs were cultured for 5 days *ex vivo*, and images were taken at 30 min intervals (S2 Video). Based on the epithelial tissue shape and the cell proliferation pattern, we classified the bell-shape formation into four transition phases consistent with the *in vivo* development (Fig 1E, S1 Fig and S2 Video). In the bud stage, the dental epithelium invaginated toward the underlying mesenchyme (phase 1, 0 to 15 hours *ex vivo* culture, equivalent to E13.0 to E13.5 *in vivo* development). In phase 2 (15 to 25 hours *ex vivo* culture, E13.5 to E14.0 *in vivo*), growth arrest was briefly observed in the mesenchyme located at the opposite side of the invaginated epithelium. The growth-arrested primary EK formed at the epithelial tip in the early cap stage. The distal portion of the invaginated epithelium then became flattened (S2 Video). In phase 3 (25 to 50 hours *ex vivo* culture, E14.0 to E15.0 *in vivo*), the epithelium adopted the cap-shaped morphology according to the invagination of the epithelial cells surrounding the primary EK. Subsequently, in phase 4 (50 to 100 hours *ex vivo* culture, E15.0 to E16.5 *in vivo*), the cervical loop, which is formed at the tip of the invaginating epithelium, continuously invaginated and elongated into the mesenchyme. The tooth germ epithelium then formed the bell-shaped morphology. During secondary EK formation, the primary EK moved to the buccal side of the secondary EK, and a new EK appeared in the enamel epithelium on the lingual side (S2 Video). The high proliferative potential of epithelial cells is maintained in the growing apex of epithelium (GAE) and contributes to the elongation of the epithelium for root formation. We found that the morphological changes and developmental timing observed in the *ex vivo* experiments are consistent with those that occur during *in vivo* development (Fig 1A and 1E). In these analyses, we were able to observe the consecutive shape changes during the transitional period from the bud to the bell stages, and the cell behaviors were visualized at single-cell resolution and associated with cell cycle progression based on the Fucci probe.

### Deformation dynamics of the developing tooth epithelium

**Fig 2:**
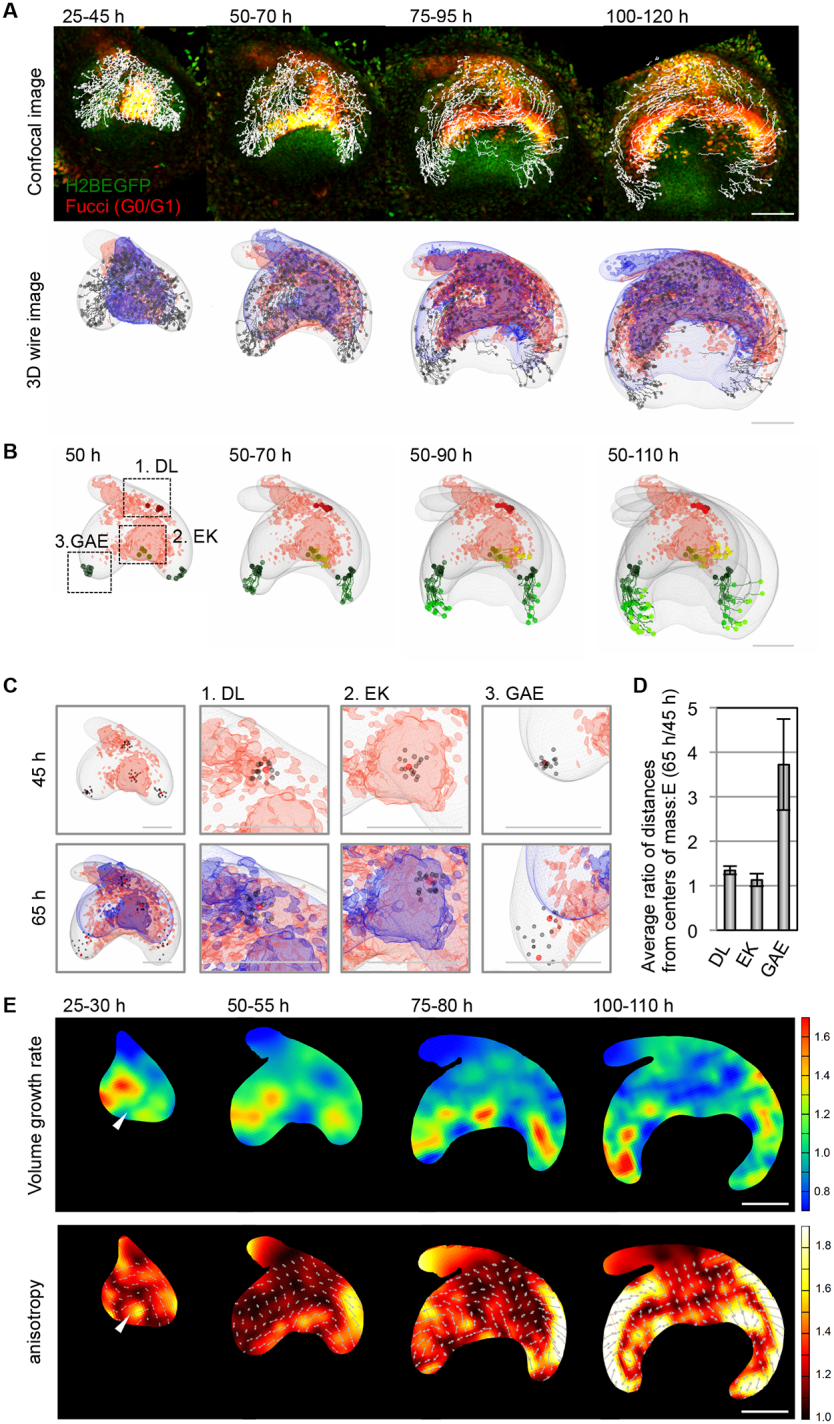
The quantitative kinetic analysis of tooth morphogenesis. **(A)** Trajectories of epithelial cells over 20 hours (h) are shown in fluorescent images (upper panel) and wire frames (lower panel) at each time point of the long-term live image. The contours of the epithelium before and after 20 hours are shown in blue and grey wire frames, respectively. The growth-arrested regions are shown in red wire frame. **(B)** Trajectories of epithelial cells in parts (EK, enamel knot; DL, dental lamina; GAE, growing apex of epithelium) of the tooth germ epithelium during tooth development. **(C)** The changes in the relative positions of cells in parts of the tooth germ epithelium. The red and gray spots indicate the center cells and surrounding cells, respectively. **(D)** Measurements of the relative distances of the epithelial cells in each region. The numbers of spots for the calculations in each region were as follow: *n* = 17 cells (DL), *n* = 16 cells (EK), *n* = 14 cells (GAE). The error bars indicate the standard errors. **(E)** Deformation analysis of the epithelial tissue for 5–10 hours. The upper and lower panels show the spatial patterns of the volume growth rate and anisotropic tissue stretching, respectively. In the lower panels, the colors indicate the degree of anisotropy, and the arrows indicate the major axes of tissue stretching. The numbers of spots used to estimate the deformation map for each time intervals were as follows: *n* = 425 cells (25–30 hours), *n* = 552 cells (50–55 hours), *n* = 532 cells (75–80 hours), *n* = 615 cells (100–110 hours). The scale bars represent 100 μm.

To quantify the complex cell behaviors during tooth morphogenesis, we tracked the 3D trajectories of the epithelial cells and mapped them onto the tooth morphology data that were reconstructed using the Imaris software (Fig 2A, S2 Fig and S3 Video). As shown in Fig 2B–2D, the degree of change of the relative positions among neighboring cells depended strongly on sub-regions within the tooth germ. In the regions in which the cell cycle was arrested, such as the dental lamina and the primary EK, the cells moved close together, whereas in the highly proliferating GAE, the cells moved apart at a faster rate. The relative positional change at each position reflected the deformation dynamics of the local tissue pieces.

To analyze the spatial-temporal patterns of tissue deformation in detail, we constructed tissue deformation maps over a 5-hour period during the stages corresponding to E13.5 to 16.5 based on the 3D cell trajectory data by applying a recently developed statistical method [24]. We then calculated the volumetric growth rate and the deformation anisotropy (biased stretching), which are typical quantities that are used to characterize tissue deformation dynamics, and illustrated their spatial-temporal patterns as heat maps (Fig 2E). During the bud-to-cap formation process (from 25 to 50 hours), as expected from the cell-cycle pattern shown with the Fucci probe, volume growth was minimally observed in the EK, whereas the surrounding epithelial tissue exhibited a high growth rate (Fig 2E and S3 Fig). Interestingly, the spatial pattern of deformation anisotropy exhibited a negative correlation with the volume growth rate around EK formation. The anisotropy was higher in the EK, and the cell populations there were deformed along the buccal-lingual axis. This result implies that the internal pressure from the growth in the epithelium surrounding the EK, along with the mechanical constraints at the boundary between the epithelium and the underlying mesenchyme, might provide the basic mechanism for the flattening of the tooth germ along the buccal-lingual axis during cap formation.

During the formation of the bell-shape in which the invagination and directional outgrowth of the epithelium along the oral-aboral axis are of primary interest, the higher growth rate region gradually shifted to the invaginating and elongating apex of the epithelium. Additionally, higher deformation anisotropies were observed throughout the GAE regions, especially at the boundary with the mesenchymal tissue surrounding the tooth germ (specifically, the dental follicle illustrated in HE staining (S5A Fig) and the basement membrane protein Nidogen (S5B Fig), which can be differentiated into the periodontal tissues comprising the periodontal ligament, cementum and alveolar bone), which implicated the involvement of the interaction with the surrounding mesenchyme in the determination of the direction of loop elongation. To verify this hypothesis, we isolated tooth germs from E13.5, E14.5 and E15.5 embryos and treated them with dispase to separate the epithelial tissue from the mesenchyme. Consequently, the direction of the GAE in E14.5 and E15.5 but not E13.5 tooth germ was drastically changed, although it maintained its elongated shape (S5 Fig and S4 Video). This observation supports the notion that the interaction with mesenchyme is essential for GAE deformation. Therefore, the quantitative analysis of the tissue deformation dynamics suggests that the precise regulation of the spatial-temporal pattern of tissue growth and the interaction with the surrounding mesenchyme are important for determining tooth germ morphology.

### Cellular mechanisms for anisotropic tissue elongation

**Fig 3:**
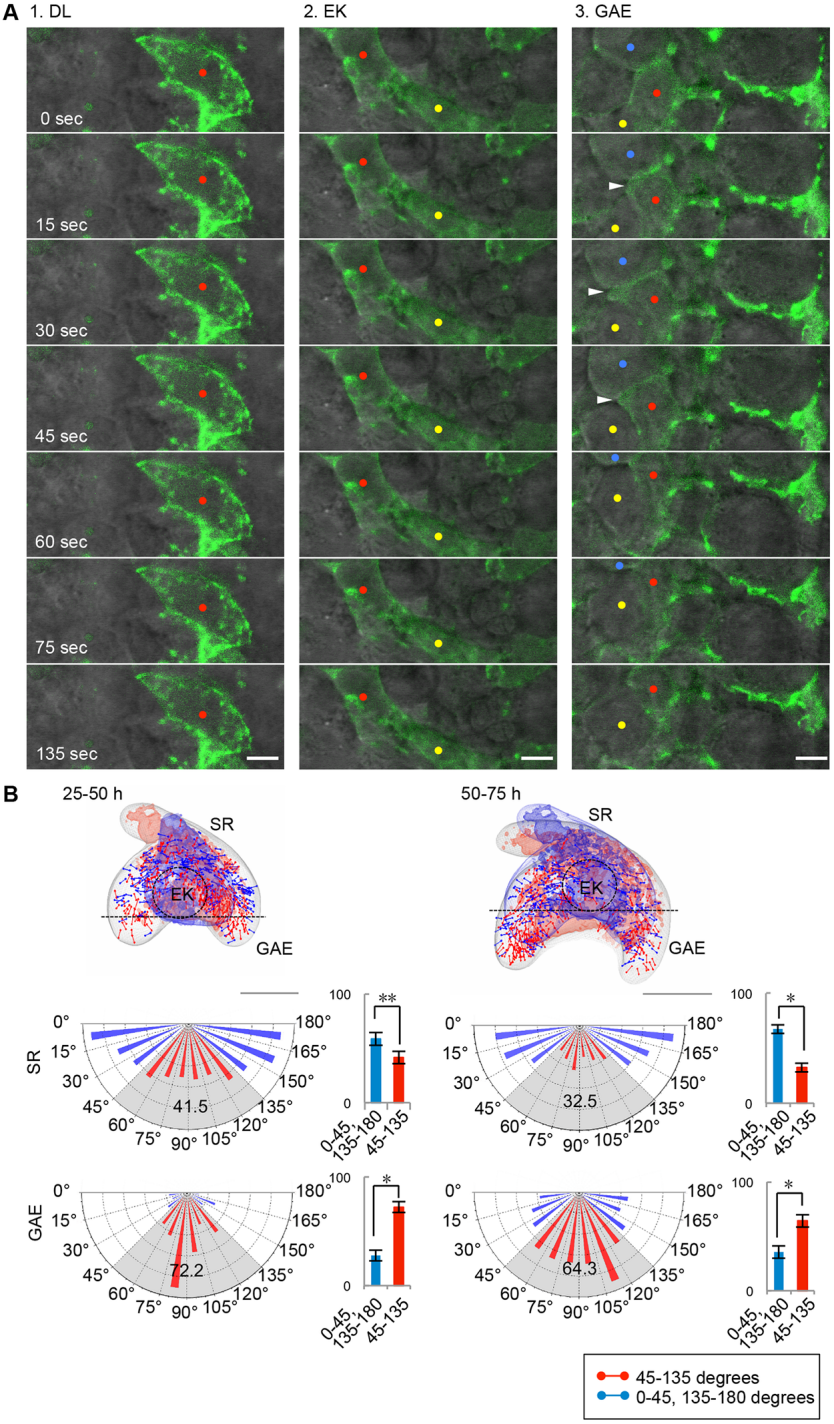
Coordinated behaviors of the epithelial cells during tooth morphogenesis. **(A)** Time-lapse sequences of the dental lamina (DL), enamel knot (EK) and growing apex of the epithelium (GAE) regions showing cell motility. The cells randomly expressed transgenes (YFP-Actin; green). The red, yellow and blue dots indicate individual cells in each region. The arrowhead indicates a membrane protrusion extending from the cell in the GAE. The scale bars represent 5 μm. **(B)** Distribution and measurement of mitotic spindle orientation in the developing tooth germ. The locations and orientations of the mitotic spindles of the dividing cells are shown in the wire frames. The mean distributions of the mitotic spindle angles (*θ*) in stellate reticulum (SR; upper) and growing apex of the epithelium (GAE; lower) are shown in the pie chart graph. The results are shown as the mean ± s.d. of three samples (*n* = 557, 627 and 626 cells [SR], *n* = 263, 183 and 234 cells [GAE]). *P < 0.01, * * 0.001 < *P* < 0.005, analyzed with *t*-tests. The scale bars represent 100 μm.

Next, we sought to identify which cellular processes generate the anisotropic tissue elongation in the growing apex during bell-shape formation. We first analyzed cell motility in the GAE with live imaging over several minutes and compared the images to those of the EK and dental lamina (DL) regions (Fig 3A and S5 Video). To visualize the individual cell-boundary line, yellow fluorescent protein-tagged actin (YFP-actin) was randomly transfected into cells in each region. Although the cell motilities in the growth-arrested DL and primary EK regions were low, in the highly proliferative GAE, motility was high and involved dynamic movements of the membrane protrusions. These observations indicated that cell motility is also different in each of the sub-regions exhibiting different rates of proliferation.

Recent studies have demonstrated that the regulation of the mitotic spindle orientation is also essential for controlling the tissue shape changes and determining the direction of elongation [22, 27]. Thus, we next analyzed the bias in the orientation of cell division during tooth morphogenesis and investigated whether the asymmetric elongation of the tooth germ epithelium relies on cell division orientation. We compared the angles of mitotic spindle orientation at different locations in the tooth germ epithelium along the oral-aboral axis using the 4D cell tracking system. In the stellate reticulum region (SR), 58.5 and 67.5% of the mitotic spindles were distributed laterally (*θ* = 0° to 45° or 135° to 180°) at 25–50 hours and 50–75 hours, respectively, in the 3D organ culture (Fig 3B and S6 Fig). In contrast, 72.2 and 64.3% of the mitotic spindles in the GAE were parallel to the oral-aboral axis (*θ* = 45° to 135°) at 25–50 hours and 50–75 hours, respectively, in the 3D organ culture (Fig 3B and S6 Fig). These statistical analyses revealed that the distribution of the mitotic orientation is significantly biased along the direction of elongation in the epithelium, which suggests the possibility that regionally oriented cell division could contribute to the determination of epithelium elongation and germ enlargement.

### Regulation of cell growth and motility via actin remodeling

**Fig 4:**
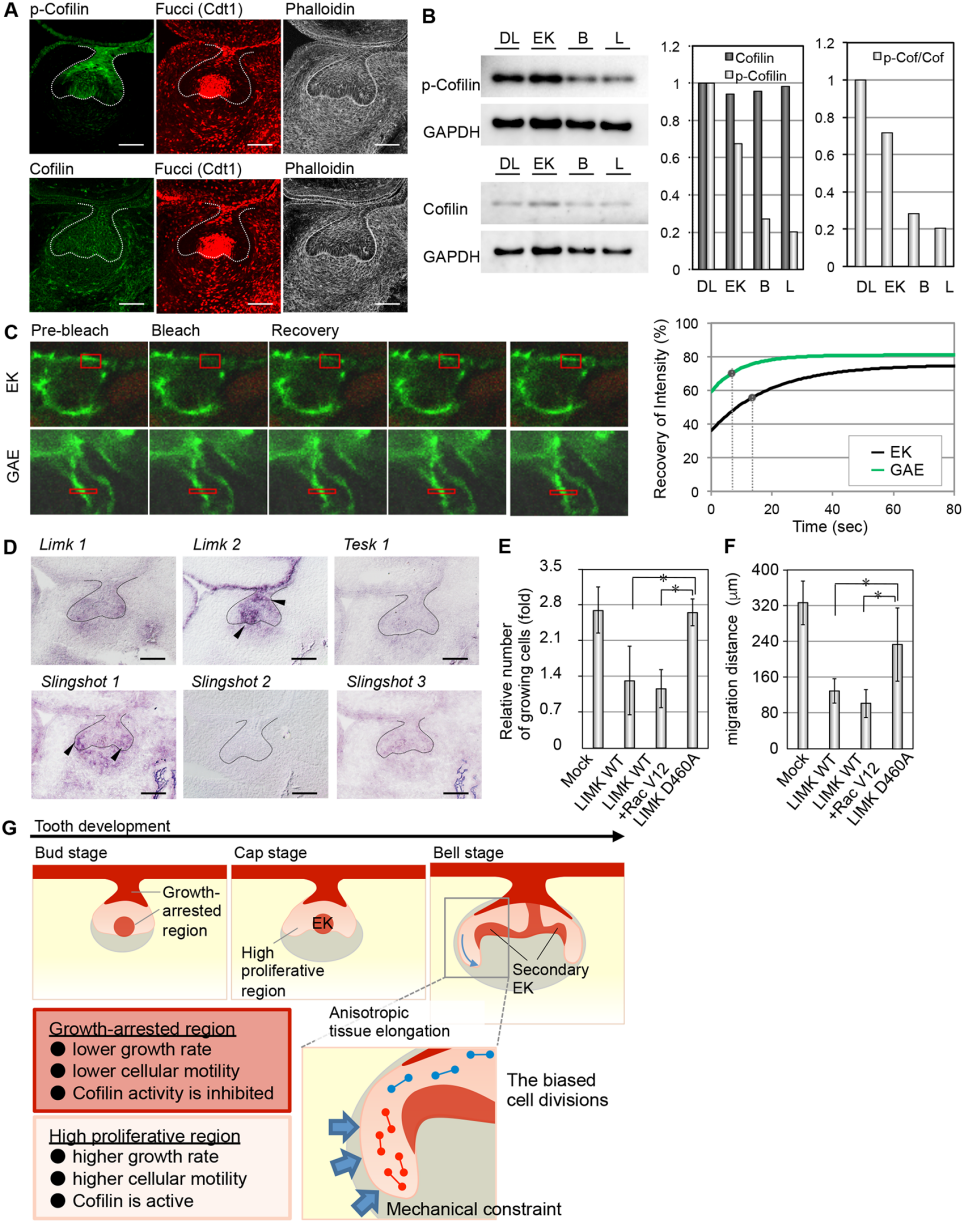
The regulation of the tooth morphogenesis via actin reorganization. **(A)** The localizations of p-cofilin (green, left upper), cofilin (green, left lower), and F-actin (white, right) were detected by immunohistochemistry. The G0/G1 phase cells (red, center) are visualized with a Fucci probe. The lingual side is on the left in all panels. **(B)** Estimations of the p-cofilin/cofilin ratios in parts of the tooth germ epithelium (EK, enamel knot; DL, dental lamina; B and L, buccal side and lingual sides of the growing apex of the epithelium). The relative amounts of cofilin and p-cofilin in the regions of the epithelium were determined by immunoblotting (left). The intensities of the bands were calculated and are indicated in the bar graphs (right). **(C)** Measurements of actin dynamics in the epithelium using fluorescence recovery after photobleaching (FRAP). The right graph illustrates the best-fit curves of the normalized fluorescence intensity during the FRAP assay. The spots indicate the half-recovery times. **(D)** Gene expression of upstream molecules that regulate cofilin activity in the E14.5 tooth germ. The lingual side is on the left in all panels. The scale bars represent 100 μm. **(E)** Inhibition of cell proliferation by cofilin phosphorylation in a WST-8 assay. The results are presented as the mean ± s.d. of triplicate experiments. *P < 0.01, analyzed by *t*-test. LIMK WT, wild-type LIM-kinase (LIMK); LIMK D460A, dominant negative LIMK mutant; Rac V12, dominant active mutant of Rac1. **(F)** Inhibition of cell migration by cofilin phosphorylation in a wound healing assay. The bars indicate the migration distances of the epithelial cells. The results are presented as the mean ± s.d. of triplicate experiments. *P < 0.01, analyzed by *t*-test. **(G)** Schematic summarizing the observed results in this study.

Cell migration, shape formation and cell proliferation are regulated by cell cytoskeletal dynamics and reorganization [6, 28–30]. We therefore investigated the involvement of cytoskeleton remodeling in the regional regulation of cell proliferation and movement in tooth morphogenesis. We first examined the spatial localizations of the cytoskeletal components and their regulatory molecules, such as small GTPases, guanine nucleotide exchange factors (GEFs) and actin binding proteins. We found that phosphorylated cofilin (p-cofilin), which is a member of the ADF/cofilin family, was localized in the growth-arrested primary EK and DL regions of the tooth germ (Fig 4A, S7A Fig). P-cofilin also localized to the corresponding regions in the hair follicles (S7B Fig), although the total cofilin was distributed uniformly in both the tooth germs and hair follicles (Fig 4A and S7 Fig). We also found that *LIM-kinase 2* (*Limk2*) but not *Limk1* or *Testis-Specific Kinase 1* (*Tesk1*, a member of the LIM kinase family that phosphorylates members of the ADF/cofilin family) was expressed at relatively high levels in the DL and EK in which growth was arrested. In contrast, the cofilin phosphatase *Slingshot 1* was localized in the GAE, where the proliferation rate was high (Fig 4D). *Slingshots 2* and *3* did not exhibit biased expression patterns in the tooth epithelium. The relative amounts of p-cofilin and the ratio of p-cofilin/total cofilin were detected by western blot analysis using laser microdissection. Both of these values were significantly higher in the DL and EK growth-arrested regions than in the GAE on both the buccal and lingual sides (Fig 4B and S8A Fig), which suggested that actin reorganization is mediated regionally through the regulation of cofilin activity in the tooth epithelium.

We subsequently examined the actin cytoskeletal remodeling in each tooth germ region by measuring the turnover of actin filaments using YFP-Actin with fluorescence recovery after photo bleaching (FRAP). We compared the fluorescence recoveries between the EK and GAE, and we found that fluorescence recovery in the bleached regions occurred more rapidly in the GAE than in the EK (Fig 4C, S8B Fig). We calculated the fluorescence decay constant (k) and fluorescence recovery half-time (t_1/2_) by fitting the plotted curve to an exponential curve (Fig 4C and S8B Fig). The actin filament turnover (t_1/2_) in the GAE (6.66 sec) was relatively high compared to that in the EK (13.33 sec). These results correlated with the cofilin phosphorylation levels.

We next investigated whether cofilin phosphorylation is involved in cell proliferation and cell motility in tooth germ-derived epithelial cells. The expression of wild-type LIM-kinase (LIMK WT), which induces cofilin phosphorylation, and/or Rac V12, which promotes LIMK activity, inhibited both cell proliferation and the migration of the primary dental epithelial cells, whereas these processes were not inhibited in the LIMK mutant LIMK D460A (Fig 4E, 4F and S8C Fig). These result suggest that signaling through the LIM-kinase (LIMK)/cofilin pathway, which functions in the growth-arrested regions, inhibits both cell proliferation and migration in the tooth germ epithelium.

## Discussion

In this study, we demonstrated that the spatiotemporal patterns of growth, division, and motility are precisely controlled during the reshaping of the tooth epithelium from the cap to the bell stages using our 4D analysis of long-term imaging of the developing tooth germ. We also found that the spatial-temporal actin rearrangement mediated by cofilin was strongly correlated with the sub-regions that exhibited the differences in cell behaviors within the epithelium over time and space during tooth morphogenesis.

The processes by which dynamic cell behaviors are orchestrated and drive organ morphogenesis have largely remained unresolved. Innovations in super-resolution imaging techniques and the development of unique fluorescent probes enabled us to visualize dynamic cellular behaviors at single-cell resolution [16, 23, 31–35]. Recent studies have demonstrated that concerted 3D (i.e., 2D space on the epithelial sheet in addition to time) cell behaviors, such as cell movement, cell division and apoptosis, contribute to correct tissue morphogenesis [18, 20, 36–38]. However, few reports have systematically quantified and analyzed the complexity of cell movements in 4D (i.e., 3D space and time) during organ development due to the difficulty of visualizing inside the tissue. In our present study, we combined novel imaging techniques and fluorescent probes and succeeded in the long-term live imaging of the developing tooth germ via the development of a 4D cell analysis system. In this system, the morphological changes and developmental timing exhibited by the *ex vivo* explants were highly consistent with those demonstrated in *in vivo*, at least in the early stage. This system enabled us to obtain precise quantitative data about cell behaviors, such as cell trajectories and cell division orientations, in three-dimensionally growing tissue at a single-cell resolution in addition to obtaining data about the dynamic changes in tooth germ morphology. These data also allowed the quantitative analysis of tissue deformation dynamics, through which we were able to link cell behaviors with organ morphology. Thus, our methods will bridge the gap between the micro- and macro-scales (i.e., the cell-to-tissue levels) and provide insights into the spatially heterogeneous patterns of cell dynamics in tissues that lead to 3D morphological changes.

Among many organs, including tooth germs and hair follicles, the morphological change from the bud to the cap-shaped epithelium is considered to be a critical step in organ morphogenesis [13, 14]. In the bud-to-cap transition during tooth morphogenesis, the EK appears at the tip of the bud epithelium where the epithelial folding is initiated. In mutant mouse embryos lacking an EK, such as the embryos of Lef1-, Msx1-, Pax9- and Pitx2-knockout mice, tooth development halts at the bud stage, which suggests that EK formation and function are essential for the transition from the bud to the cap stage [14]. Previous studies have presumed that signaling molecules secreted from the EK generate differential growth in the tooth germ by stimulating the proliferation of surrounding cells, including mesenchymal cells, and consequently induce the deformation of the epithelium [13]. That hypothesis is based on histological analysis due to the lack of other methods. In this study, we demonstrated the cell behaviors and cell cycle progression during tooth morphogenesis with live imaging using the Fucci probe. As previously reported, the cell cycle clearly differs between the EK and its surrounding epithelium. The quantitative tissue deformation analysis demonstrated that the spatial pattern of the volume growth rate at the tissue level was consistent with that of the cell cycle. Thus, it is certain that explosive cell proliferation serves as the main driving force of tissue expansion throughout tooth morphogenesis. However, the precise shape of the tooth epithelium cannot be determined only by cell proliferation. Deformation anisotropy, another characteristic of tissue deformation dynamics, is the important factor of tissue shaping, and it exhibited a trend opposite to that of the volume growth rate. In particular, higher anisotropy along the buccal-lingual axis was observed around EK in the bud stage and that along the oral-aboral axis in the GAE regions in the bell stage. Those reflect the tissue flattening and elongation, respectively. As verified by an epithelium isolation experiment, the spatial-biased anisotropy implicates that epithelial bending occurs in the presence of the interaction with the surrounding dental mesenchyme via the extracellular matrix, although it is unknown whether the mesenchyme provides spatial constraints or the foothold for epithelial cell migration and attachment (Fig 2C, S5 Fig and S4 Video). The possibility that the surrounding mesenchyme serves as a mechanical constraint to the bud-to-cap deformation of the epithelium has also been supported recently in a theoretical model [39]. Moreover, the cell-level analysis in the present study indicates that the anisotropic tissue deformation in the GAE was due to the combination of the tip-biased cell motility and the cell division oriented along the elongation axis (Fig 4G). However, further study is still needed to determine which cell behavior is the most critical and decisive for tooth epithelium morphogenesis.

Actin filaments are crucial cytoskeletal structures that convert intracellular and extracellular information into physical and mechanical forces. Actin filaments participate in the dynamic regulation of multiple cellular functions, such as shape changes, cell adhesion, intercellular transport, cell movement and cell division, by providing the driving forces for these processes [7, 28, 40]. These actin filament functions are supported by ADFs that mediate normal actin reorganization [41]. Although studies on Drosophila and vertebrates have provided evidence that the mechanical tension induced by actomyosin is essential for cell shape changes and rearrangements during epithelial folding and polarity formation, the importance of actin reorganization in morphogenesis has remained unexplored [7, 42]. Previously, it was reported that cofilin defects cause the failure of neural tube closure, and the loss of both cofilin and destrin results in the arrest of epithelial branching with F-actin accumulation in kidney organogenesis [43, 44]. The signaling pathway related to actin organization is also known to be related to cell cycle progression in addition to providing the machinery and force for cell division and migration [45]. Indeed, LIMK has been reported to inhibit the cell cycle progression at the G1/S phase [29, 30]. Based on these studies, the LIMK/cofilin pathway has been suggested to be associated not only with cell shape changes and cell migration but also with the G1/S phase arrest that underlies epithelial morphogenesis. In our current study, we demonstrated that actin organization was inhibited in the low proliferative tooth germ EK, which expressed LIMK, and cell motility was also suppressed. These results suggest that the LIMK/cofilin signaling pathway affects cell motility and proliferation and consequently regulates the spatial-temporal orchestration of cell behaviors during tooth morphogenesis.

In conclusion, our findings based on our 4D cell analysis system shed light on how the multicellular events in tooth morphogenesis are controlled across space and time. We propose the possibility that cell behaviors in the epithelium during organogenesis are orchestrated through the LIMK/cofilin pathway. Further studies revealing the essential parameters for the regulation of individual cell behaviors and tissue formation will improve the understanding of organ morphogenesis as a system and facilitate a comprehensive understanding of organ morphogenesis.

## Supporting Information

S1 Fig Tooth morphogenesis and spatial-temporal cell proliferation (related to Fig 1). Frontal sections of the mandibular molar tooth germ derived from Fucci transgenic mice at E13.5–18.5. Hoechst (upper), Fucci fluorescence (middle), and merged (lower) images are shown. The lingual side is on the left in all panels. The scale bars represent 100 μm.
(TIF)

S2 Fig The quantitative kinetic analysis of tooth morphogenesis (related to Fig 2). **(A)** Photographs showing the cutting surface region (z < 30 μm, upper panels) and the deeper region (z > 30 μm, lower panels) in *ex vivo* cultured tooth germ. The white arrowheads indicate mitotic cells. **(B)** The relative numbers of cell divisions per tissue volume in the surface area and the deeper analyzed area. **(C)** Schematic of the quantitative kinetic analysis based on Imaris image processing. The white arrowhead in the left-lower panel indicates the borderline between the epithelium and mesenchyme. This working process was repeated at sequential time points. **(D)** The information that can be acquired with this system. **(E)** Schematics showing typical analysis results from Imaris image processing. The positions of the epithelial cells before and after 20 hours are indicated by blue and grey spots. The trajectories of individual epithelial cells over 20 hours are indicated by white lines. The contours of the epithelium before and after 20 hours are shown in blue and grey wire frames.
(TIF)

S3 Fig Deformation analysis of developing tooth epithelium (related to Fig 2). **(A)** The trajectories of epithelial cells over 5 hours are shown on fluorescent images (upper panel) and wire frames (lower panel) at each time point of the long-term live imaging. The scale bars represent 100 μm. **(B)** The raw data for the epithelial tissue deformation analysis. The upper and lower panels illustrate the changes in the epithelial shape and cell position before and after 5 hours, respectively. The green spots indicate the cell positions, and magenta grid squares indicate tissue micro-compartments. The scale bars represent 100 μm. **(C)** Deformation analysis of the epithelial tissues over 5 hours. The upper and lower panels illustrate the spatial patterns of the volume growth rates and anisotropic tissue stretching, respectively. In the lower panels, the colors indicate the degree of anisotropy, and the arrows indicate the major axes of tissue stretching. The numbers of spots used to estimate the deformation map for each time intervals were as follows: *n* = 425 cells (25–30 hours), *n* = 425 cells (30–35 hours), *n* = 485 cells (35–40 hours), *n* = 485 cells (40–45 hours), *n* = 547 cells (45–50 hours), and *n* = 552 cells (50–55 hours). The scale bars represent 100 μm.
(TIF)

S4 Fig Deformation analysis of the epithelial tissue over 25–30 hours (A) and 30–35 hours (B) at different z positions. The upper and lower panels illustrate the spatial patterns of the volume growth rate and anisotropic tissue stretching, respectively. In the lower panels, the colors indicate the degree of anisotropy, and the arrows indicate the major axes of tissue stretching. The numbers of spots used to estimate the deformation map in each z slice were as follows: *n* = 129 cells (z = 30–41 μm), *n* = 125 cells (z = 41–52 μm), *n* = 80 cells (z = 52–63 μm), and *n* = 91 cells (z = 63–74 μm).
(TIF)

S5 Fig The epithelial tissue elongation direction is subject to spatial restriction by the surrounding mesenchyme. **(A)** Histological analysis of the E14.5 molar tooth germ. The yellow dashed line indicates the border between the tooth germ mesenchyme and the oral mesenchyme. The scale bars represent 100 μm. E, epithelium; M, mesenchyme; DF, dental follicle. **(B)** Tooth germ mesenchymal cells condense around the epithelium. The nuclei (blue) and Nidogen (red) were detected by immunohistochemistry. The scale bars represent 100 μm. E, epithelium; M, mesenchyme; DF, dental follicle. **(C)** Observation of the epithelial shape changes the epithelium and mesenchyme were separated by enzyme treatment. E13.5 (upper panel) and E14.5 (middle panel) and E15.5 (lower panel) were used. The lingual side is on the left in all panels.
(TIF)

S6 Fig The mitotic spindle angles correlate well with the elongation direction of the epithelial tissue (related to Fig 3). The graphs represent the distributions of the mitotic spindle angles (*θ*) in the stellate reticulum and the growing apex of the tooth germ epithelium over 30–50 hours (white bar) and 50–70 hours (black bar). The error bars show ± s.d. of three samples.
(TIF)

S7 Fig Cofilin activity is regulated spatiotemporally during tooth development (related to Fig 4). **(A)** The localizations of p-cofilin, cofilin and F-actin in the tooth germ were detected by immunohistochemistry at E13.5–16.5. G0/G1 phase cells (red, center) are visualized with the Fucci probe. The lingual side is on the left in all panels. The scale bars represent 100 μm. **(B)** Phosphorylated cofilin was localized not only in the tooth germs (left two columns) but also in the hair germs (right two columns). The scale bars represent 100 μm.
(TIF)

S8 Fig Cofilin activity is regulated spatiotemporally during tooth development (related to Fig 4). **(A)** The acquisition areas of the tooth germ epithelium used for the immunoblotting analysis are illustrated. **(B)** Measurement of the actin dynamics in the epithelium using fluorescence recovery after photobleaching (FRAP). The raw data are shown with the best-fit curves of the normalized fluorescence intensities in the graph. The spots indicate the half-recovery times. The results are shown as the mean ± s.d. of five samples. **(C)** Inhibition of cell migration by cofilin phosphorylation in a wound-healing assay. Phase contrast images are shown. The scale bars represent 100 μm.
(TIF)

S1 Video *Ex vivo* time-lapse tooth germ imaging system. The molar tooth germ was reconstructed three-dimensionally from frontal sections of the tooth germ derived from a transgenic mouse embryo co-expressing Fucci-G0/G1 probe and H2BEGFP. The molar tooth germs were cut into 250–300 μm-thick frontal slices and then cultured on a glass-bottomed dish for *ex vivo* live imaging.
(MP4)

S2 Video *Ex vivo* live imaging of a developing tooth germ. Time-lapse imaging of a developing tooth germ derived from a transgenic mouse embryo co-expressing Fucci-G0/G1 probe and H2BEGFP. The tooth germ was cultured for 5 days *ex vivo*, and the images were taken at 30-min intervals. The lingual side is on the left. Also see Fig 1E.
(MP4)

S3 Video Quantitative kinetic analysis of epithelial morphogenesis. The trajectories of epithelial cells over 20 hours acquired from quantitative kinetic analysis are illustrated with raw live imaging data (left) and a wire frame (right). The lingual side is on the left. Also see Fig 2A.
(MP4)

S4 Video Time-lapse recording of epithelial shape changes following treatment with dispase. Time-lapse recording of epithelial shape changes when an E14.5 tooth germ was treated with dispase. The lingual side is on the left. Also see S5 Fig.
(MP4)

S5 Video Time-lapse recording of the DL, EK and GAE regions. Time-lapse recording of the DL, EK and GAE regions showing cell motility. Also see Fig 3A.
(MP4)
