# Supplementary figures and images for: Coordination of Cellular Dynamics Contributes to Tooth Epithelium Deformations
Source: PLoS One. 2016 Sep 2;11(9):e0161336. doi: 10.1371/journal.pone.0161336 (PMC5010284; doi:10.1371/journal.pone.0161336)

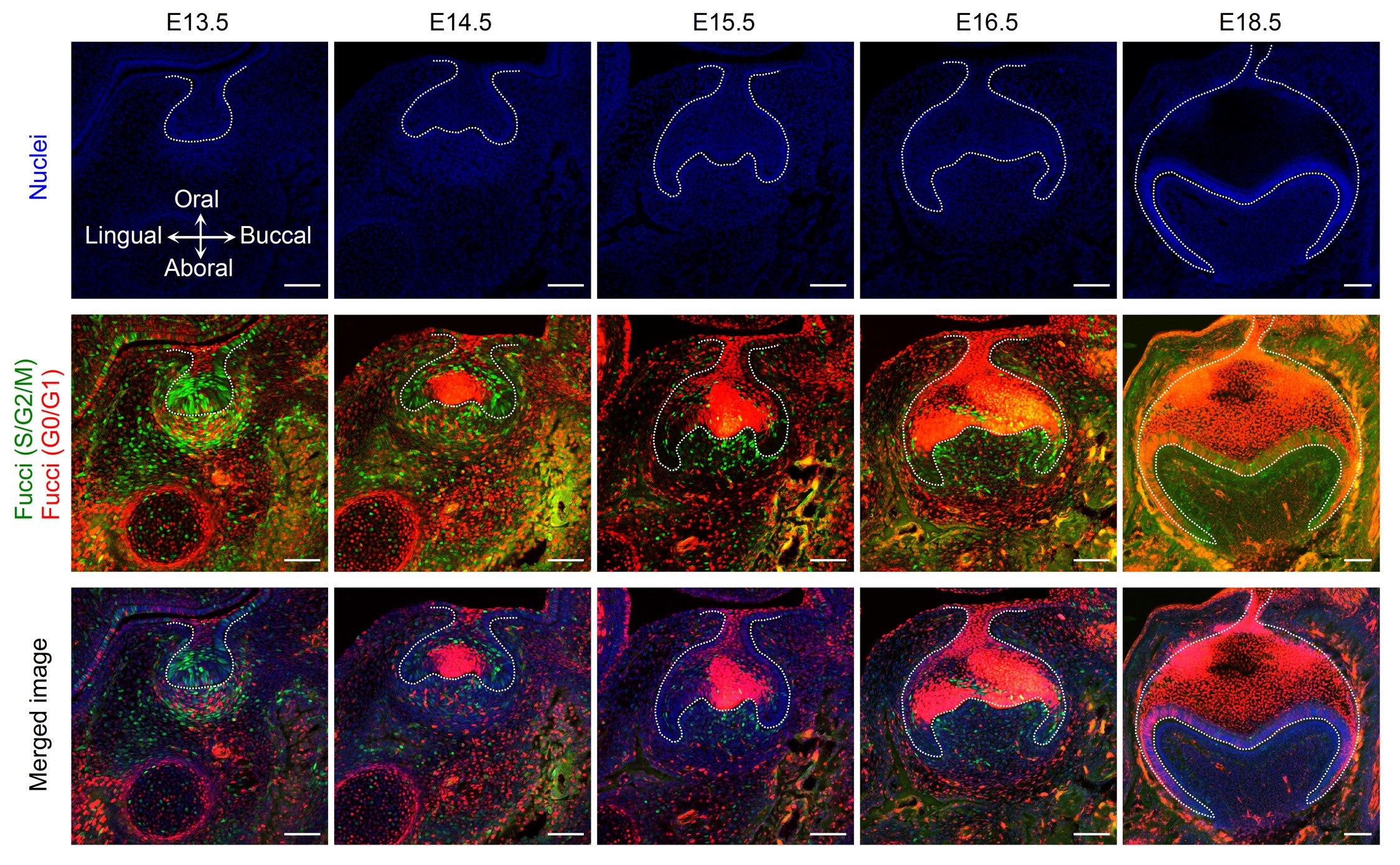

Supplement: S1 Fig — Frontal sections of the mandibular molar tooth germ derived from Fucci transgenic mice at E13.5–18.5. Hoechst (upper), Fucci fluorescence (middle), and merged (lower) images are shown. The lingual side is on the left in all panels. The scale bars represent 100 μm. (TIF) [file pone.0161336.s001.tif]

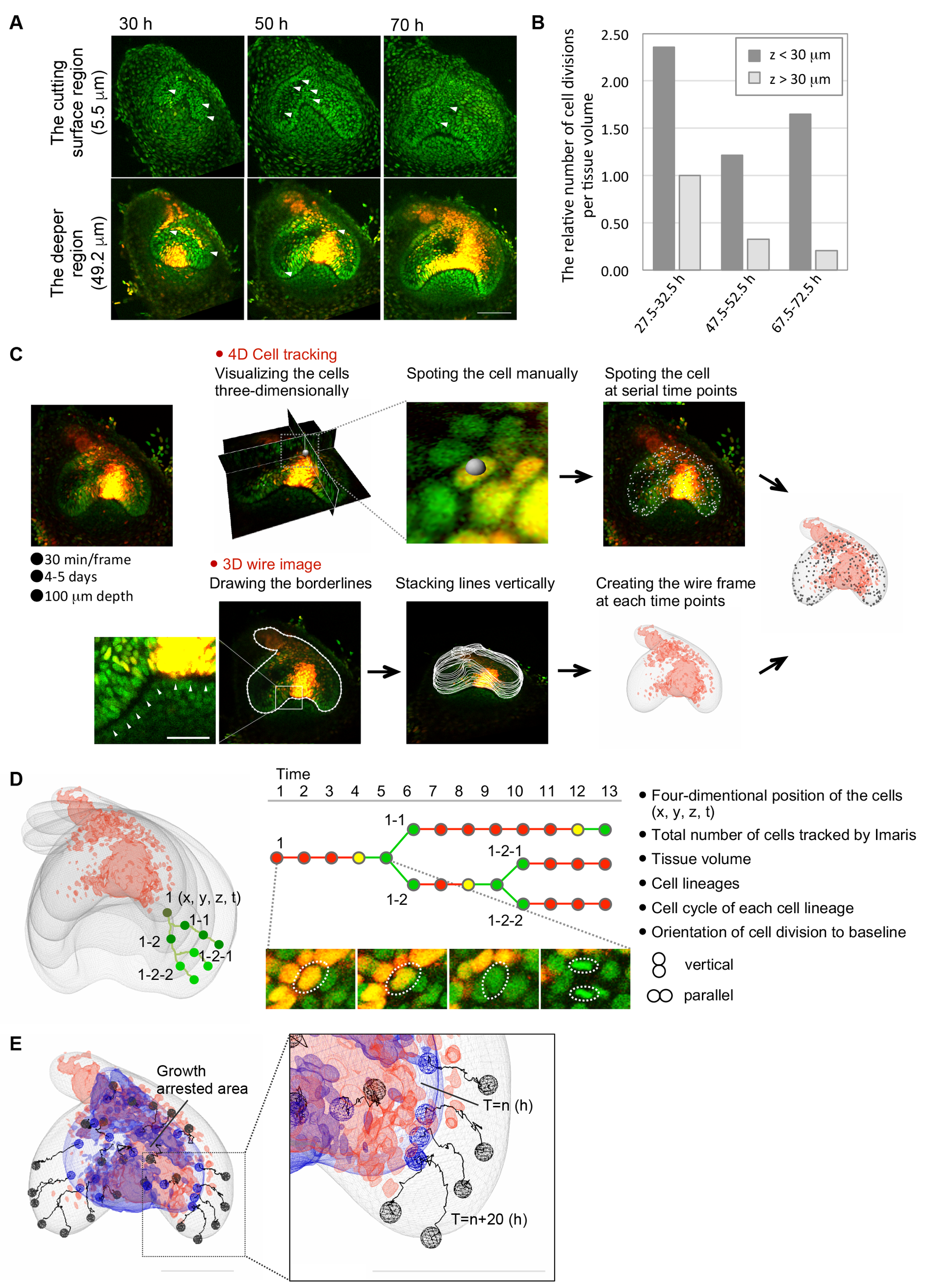

Supplement: S2 Fig — (A) Photographs showing the cutting surface region (z < 30 μm, upper panels) and the deeper region (z > 30 μm, lower panels) in ex vivo cultured tooth germ. The white arrowheads indicate mitotic cells. (B) The relative numbers of cell divisions per tissue volume in the surface area and the deeper analyzed area. (C) Schematic of the quantitative kinetic analysis based on Imaris image processing. The white arrowhead in the left-lower panel indicates the borderline between the epithelium and mesenchyme. This working process was repeated at sequential time points. (D) The information that can be acquired with this system. (E) Schematics showing typical analysis results from Imaris image processing. The positions of the epithelial cells before and after 20 hours are indicated by blue and grey spots. The trajectories of individual epithelial cells over 20 hours are indicated by white lines. The contours of the epithelium before and after 20 hours are shown in blue and grey wire frames. (TIF) [file pone.0161336.s002.tif]

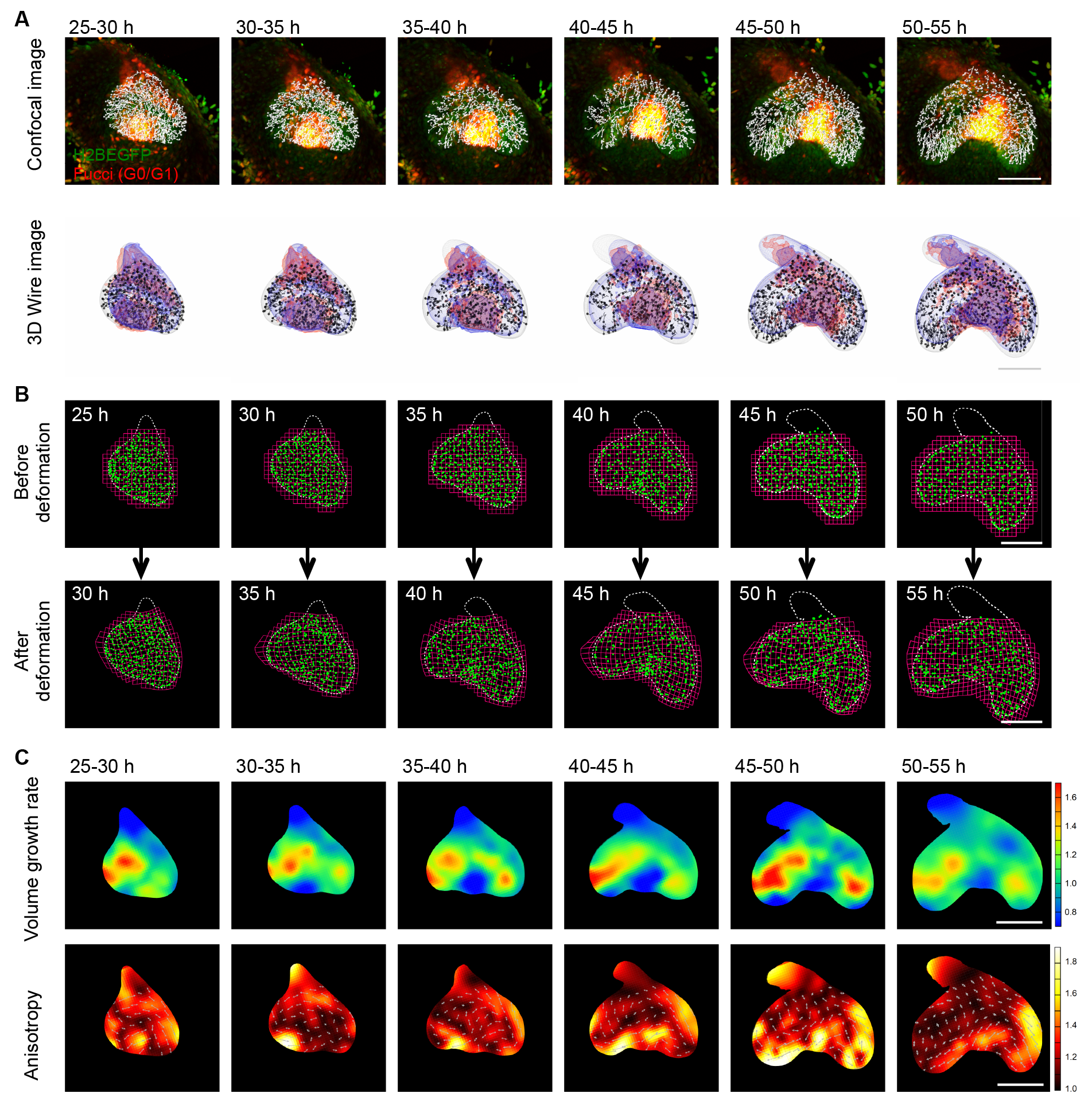

Supplement: S3 Fig — (A) The trajectories of epithelial cells over 5 hours are shown on fluorescent images (upper panel) and wire frames (lower panel) at each time point of the long-term live imaging. The scale bars represent 100 μm. (B) The raw data for the epithelial tissue deformation analysis. The upper and lower panels illustrate the changes in the epithelial shape and cell position before and after 5 hours, respectively. The green spots indicate the cell positions, and magenta grid squares indicate tissue micro-compartments. The scale bars represent 100 μm. (C) Deformation analysis of the epithelial tissues over 5 hours. The upper and lower panels illustrate the spatial patterns of the volume growth rates and anisotropic tissue stretching, respectively. In the lower panels, the colors indicate the degree of anisotropy, and the arrows indicate the major axes of tissue stretching. The numbers of spots used to estimate the deformation map for each time intervals were as follows: n = 425 cells (25–30 hours), n = 425 cells (30–35 hours), n = 485 cells (35–40 hours), n = 485 cells (40–45 hours), n = 547 cells (45–50 hours), and n = 552 cells (50–55 hours). The scale bars represent 100 μm. (TIF) [file pone.0161336.s003.tif]

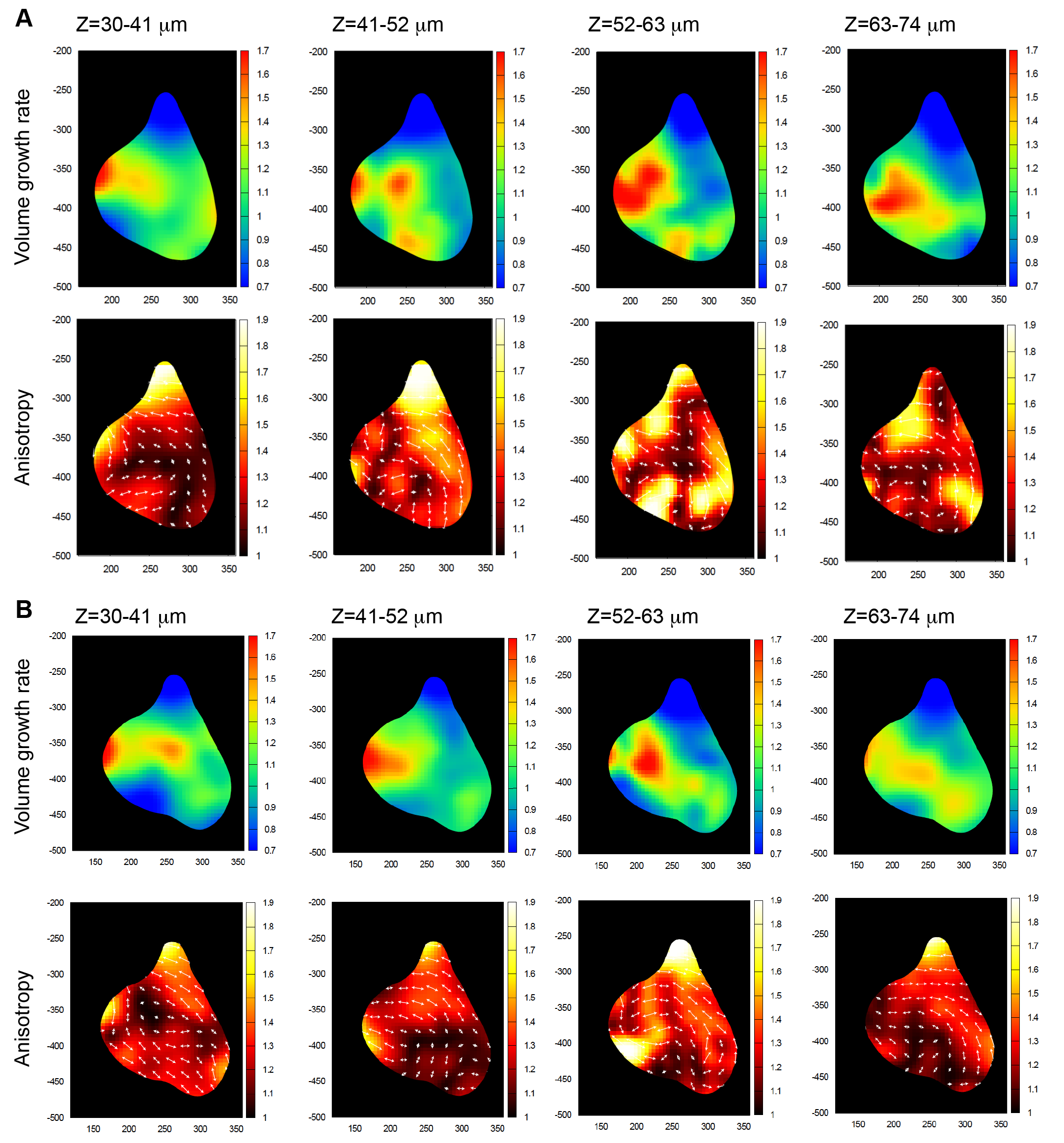

Supplement: S4 Fig — The upper and lower panels illustrate the spatial patterns of the volume growth rate and anisotropic tissue stretching, respectively. In the lower panels, the colors indicate the degree of anisotropy, and the arrows indicate the major axes of tissue stretching. The numbers of spots used to estimate the deformation map in each z slice were as follows: n = 129 cells (z = 30–41 μm), n = 125 cells (z = 41–52 μm), n = 80 cells (z = 52–63 μm), and n = 91 cells (z = 63–74 μm). (TIF) [file pone.0161336.s004.tif]

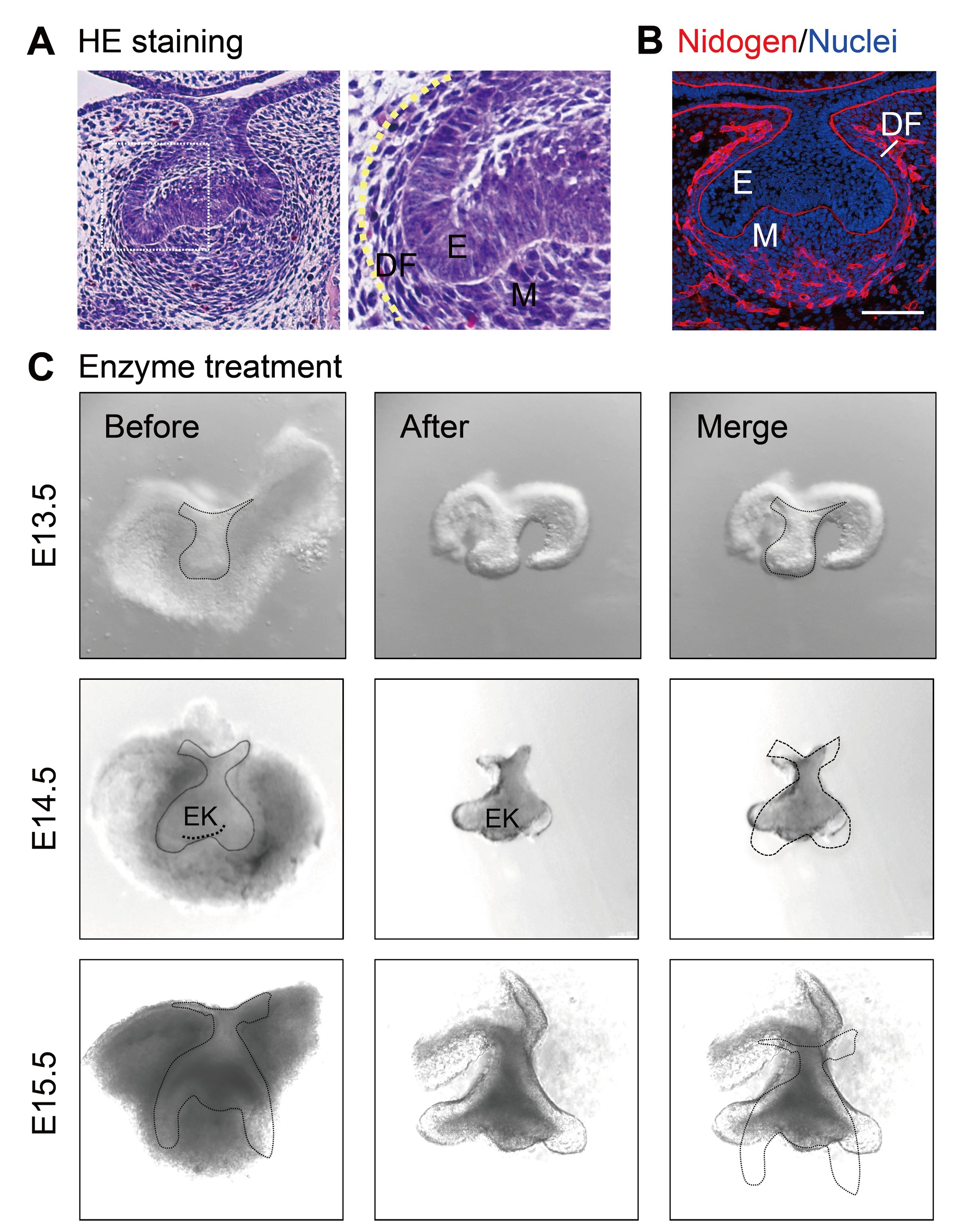

Supplement: S5 Fig — (A) Histological analysis of the E14.5 molar tooth germ. The yellow dashed line indicates the border between the tooth germ mesenchyme and the oral mesenchyme. The scale bars represent 100 μm. E, epithelium; M, mesenchyme; DF, dental follicle. (B) Tooth germ mesenchymal cells condense around the epithelium. The nuclei (blue) and Nidogen (red) were detected by immunohistochemistry. The scale bars represent 100 μm. E, epithelium; M, mesenchyme; DF, dental follicle. (C) Observation of the epithelial shape changes the epithelium and mesenchyme were separated by enzyme treatment. E13.5 (upper panel) and E14.5 (middle panel) and E15.5 (lower panel) were used. The lingual side is on the left in all panels. (TIF) [file pone.0161336.s005.tif]

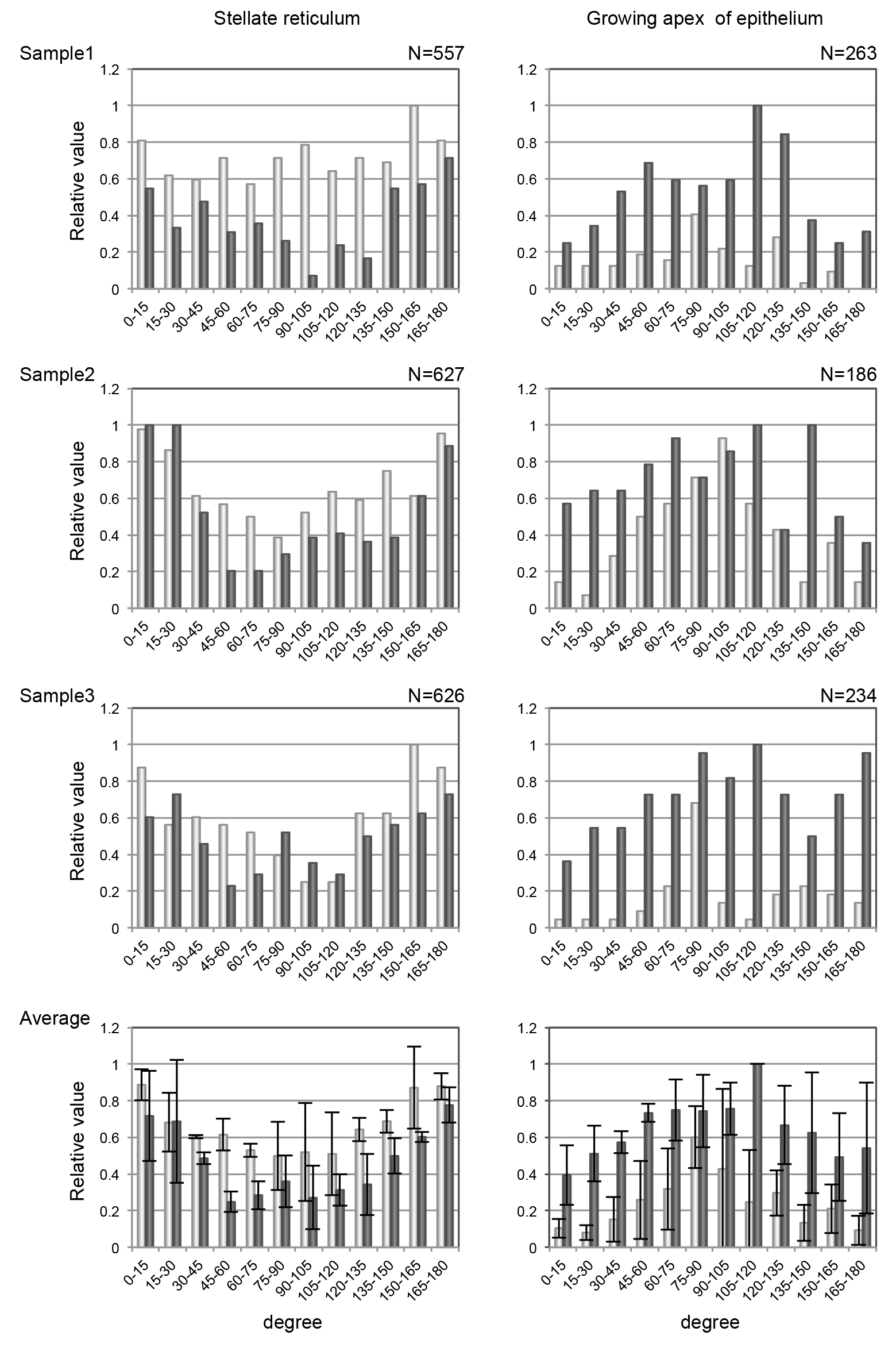

Supplement: S6 Fig — The graphs represent the distributions of the mitotic spindle angles (θ) in the stellate reticulum and the growing apex of the tooth germ epithelium over 30–50 hours (white bar) and 50–70 hours (black bar). The error bars show ± s.d. of three samples. (TIF) [file pone.0161336.s006.tif]

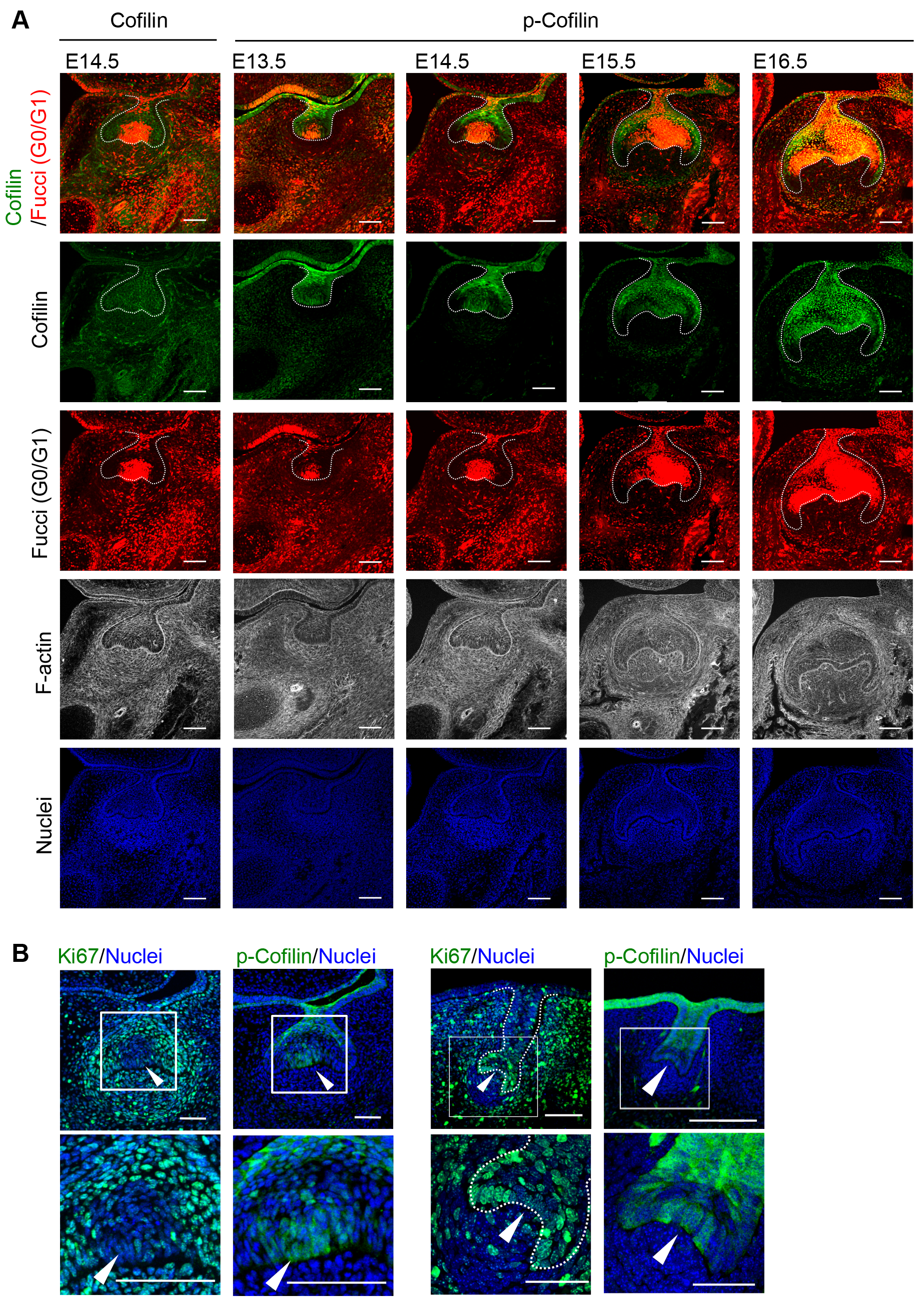

Supplement: S7 Fig — (A) The localizations of p-cofilin, cofilin and F-actin in the tooth germ were detected by immunohistochemistry at E13.5–16.5. G0/G1 phase cells (red, center) are visualized with the Fucci probe. The lingual side is on the left in all panels. The scale bars represent 100 μm. (B) Phosphorylated cofilin was localized not only in the tooth germs (left two columns) but also in the hair germs (right two columns). The scale bars represent 100 μm. (TIF) [file pone.0161336.s007.tif]

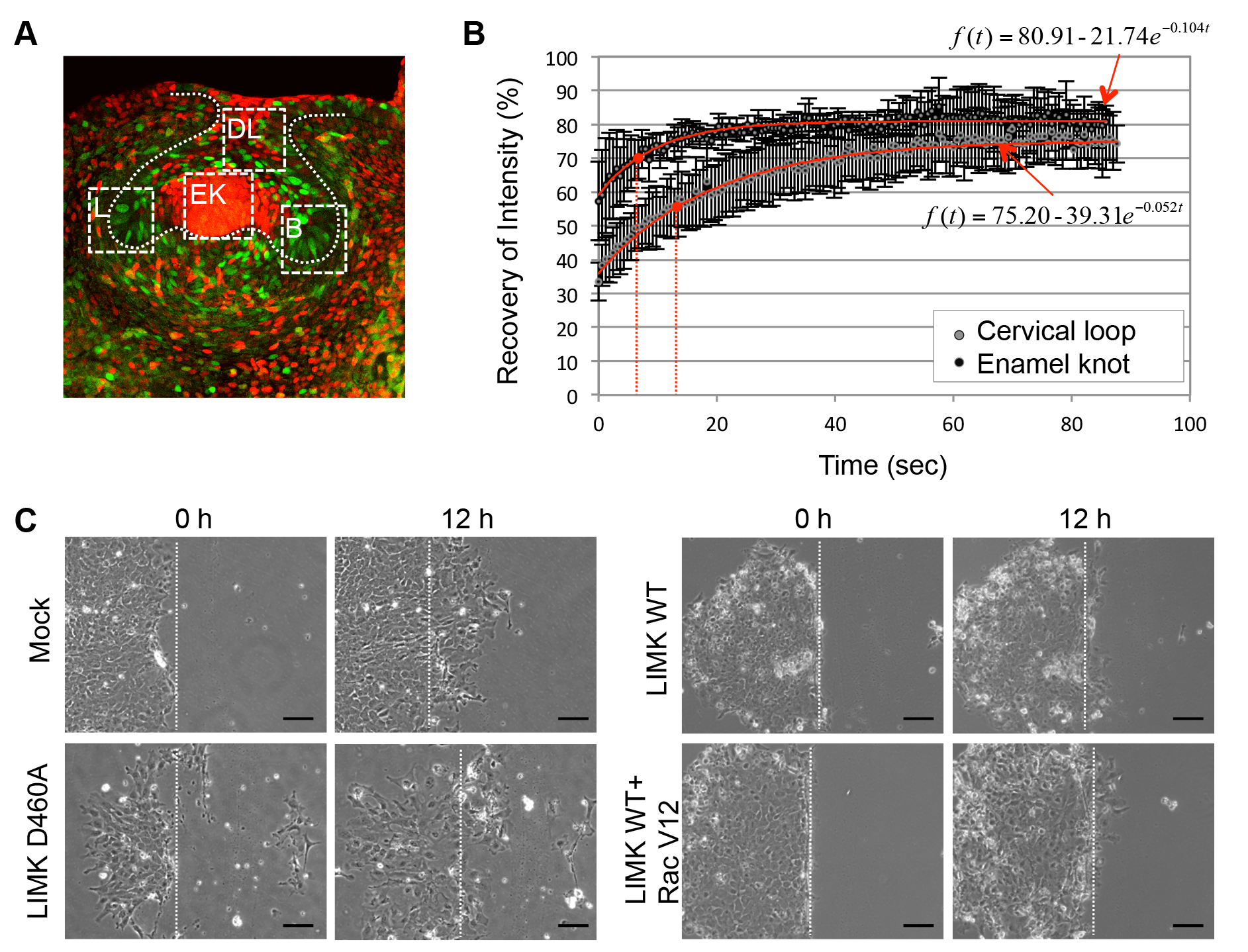

Supplement: S8 Fig — (A) The acquisition areas of the tooth germ epithelium used for the immunoblotting analysis are illustrated. (B) Measurement of the actin dynamics in the epithelium using fluorescence recovery after photobleaching (FRAP). The raw data are shown with the best-fit curves of the normalized fluorescence intensities in the graph. The spots indicate the half-recovery times. The results are shown as the mean ± s.d. of five samples. (C) Inhibition of cell migration by cofilin phosphorylation in a wound-healing assay. Phase contrast images are shown. The scale bars represent 100 μm. (TIF) [file pone.0161336.s008.tif]
